# Prevalence and Correlates of Pain in People Aged 60 Years and above in Singapore: Results from the WiSE Study

**DOI:** 10.1155/2016/7852397

**Published:** 2016-06-13

**Authors:** Pratika Satghare, Siow Ann Chong, Janhavi Vaingankar, Louisa Picco, Edimansyah Abdin, Boon Yiang Chua, Mythily Subramaniam

**Affiliations:** Research Division, Institute of Mental Health, Singapore 539747

## Abstract

*Background*. Although pain is experienced among people of all ages, there is a need to study its risk factors and impact among older adults.* Aims*. The study sought to determine the prevalence, sociodemographics, and clinical correlates of pain along with association of pain with disability among older adults in Singapore.* Methods*. The WiSE study was a comprehensive cross-sectional, single phase, epidemiological survey conducted among the elderly aged 60 years and above and used a nationally representative sample of three main ethnic groups in Singapore: Chinese, Malays, and Indians. The survey administered 10/66 protocol pain questionnaire, sociodemographic questionnaire, health status questionnaire, World Health Organization Disability Assessment Scale (WHODAS 2.0), and Geriatric Mental State (GMS) examination.* Results*. A total of 2565 respondents completed the study giving a response rate of 65.5%. The prevalence of pain among the elderly aged 60 years and above is 19.5 %. Females, incomplete primary education Indians, and those diagnosed with any chronic health condition were associated with risk of pain and disability.* Conclusion*. Study findings showed that disability related to pain among the elderly is considerable making it a priority to reduce the morbidity and disability among the elderly with pain.

## 1. Introduction

Pain is a common and a significant problem among many older adults which is often associated with worse health due to greater functional impairment, disability, depression, dementia, impaired sleep, and social isolation [1]. Pain is not an inevitable part of aging but unfortunately is often accepted as a normal part of aging among older adults for which they may be undertreated [2]. Studies have indicated that as many as 50% of older adults who live in the community and 45% to 80% of those in nursing homes suffer from this problem [3]. At any age, pain has significant impact on the life of an individual, but the effect may be greater on older adults as compared to younger adults [4].

Pain prevalence increases with increasing age among older adults [5]. Prior research demonstrated increased pain prevalence with increasing age and highlighted pain as a frequent problem for a large number of older adults [6, 7]. A systematic review suggests a peak or plateau in the prevalence of pain by age 65 and a decline in reported pain in the old-old (75–84 years), and oldest-old (85+ years) [8]. Older patients often experience pain in multiple sites, compounding pain-related suffering and disability. Pain is one of the most widely cited symptoms underlying disability among older adults and also pain is an outcome of various comorbid illnesses [9, 10]. Hence, the multidimensional impact of pain decreases the ability of older adults to effectively respond to physiologic stressors, ultimately leading to the development of frailty [5]. Cognitive impairment due to dementia, depression, and/or delirium represents a particular challenge to pain management because older adults with these conditions may be unable to verbalize their pain. The prevalence of pain among older adults with dementia varies greatly from 28% to 83% [11]. Another study observed that pain in older men was associated with increased frailty and also indicated that mood may be a more important factor in the association between pain and frailty than physical illnesses [12].

Population aging is occurring in nearly every country of the world [13]. Singapore is a country located in Southeast Asia with a multiethnic population (74.3% are Chinese, 13.3% are Malays, 9.1% are Indians, and 3.3% belong to other ethnic groups) and has one of the world's fastest aging population. Population of older adults in Singapore has grown dramatically from 2.5% in 1965 to 11% in 2014 [14]. Data on the prevalence of pain and its causative factors among older adults in Singapore is limited. Hence, this study sought to determine the prevalence and impact of pain in a large, nationally representative sample of people aged 60 years and above in Singapore. Specifically, the aims of the current study were to (1) determine the overall prevalence of pain, (2) evaluate the factors associated with pain including sociodemographic correlates and chronic diseases (hypertension, transient ischaemic attack, diabetes mellitus, dementia, and depression), and (3) assess the effect of pain on disability.

## 2. Methods

The WiSE survey was a comprehensive single phase survey conducted across Singapore with older adults aged 60 years and above. The study adopted the 10/66 Dementia Research Group protocols [15] to establish the prevalence of dementia in Singapore's elderly resident population. A cross-sectional epidemiological survey was conducted with 2565 participants who were randomly selected from a national database with information on all Singapore residents aged 60 years and above. The database consisted of administrative data (i.e., name, identification card number, ethnicity, gender, and address) of all the citizens and permanent residents in Singapore, which was updated on a regular basis. A probability sample was randomly selected using a disproportionate stratified sampling design according to ethnicity (Chinese (38.5%), Malay (30.1%), Indian (30.1%), and others (1.4%)) and age groups (60–74, 75–84, and 85 years and above) [16]. Residents in the older age group and also those belonging to Malay and Indian ethnic groups were oversampled in order to improve reliability of estimates for subgroup analysis by achieving sufficient sample size. Older adults residing in day care centres, nursing homes, and institutions were included in the study. 10/66 questionnaires were available in English, Chinese, and Tamil while the research team translated the instruments into Malay. The questionnaires were also transcribed into three major dialects, Hokkien, Cantonese, and Teochew, and choice of administered language was based on the participant's preferences. An informant selected for each participant was a “person who knew the older person best” and those informants were most commonly coresidents, family members, or caregivers of the participant [17]. Written informed consent was obtained from all participants; in the event that the respondent was unable to understand or give consent, consent was obtained from a legally acceptable representative. Trained interviewers administered the adapted 10/66 questionnaires through face to face interviews to 2565 respondents and their informants. All the respondents received the full assessment, lasting approximately 2-3 hours. A Computer Assisted Personal Interviewing (CAPI) mode was used for real-time data collection in the field. This study yielded a response rate of 65.6%. The study was approved by institutional ethics review boards, National Healthcare Group Domain Specific Review Board (DSRB) and the SingHealth Centralised Institutional Review Board (CIRB). Detailed information of the study has been provided in earlier paper [16].

### 2.1. Measurements and Assessments

The measurements and assessments are as follows:

- *Pain Questionnaire (10/66 Research Protocol, Participant Version) [15]*. During the interview, participants were asked three questions to assess frequency of pain, severity of pain, and restriction in activity due to pain. The participants were asked, “In the past month, how often has any health condition caused you pain?” and the response options available were “never,” “2-3 times in the past month,” “about once a week,” “2-3 days a week,” and “every day.” After excluding the “Never” option, for the purpose of analysis, other options were collapsed, recoded, and categorized into the following: (1) “More than once a month” including “2-3 times in the past month” and “about once a week” and (2) “more than once a week” including “2-3 days a week” and “every day.” With regard to pain severity, the question asked was, How would you rate severity of the pain in the last month? Would you say it is, “Mild pain”, “Moderate pain”, “Severe pain”, “Very severe pain”, “Unbearable pain”? Except “Mild pain” and “Moderate pain,” remaining responses, “Severe pain,” “Very severe pain,” and “Unbearable pain,” were categorized into “Severe pain” for the purpose of analysis. Restriction in doing daily activities due to pain was assessed by “When the pain is at its worst, how much does it restrict the things that you do?” The response options available were “Not at all,” “Not very much,” “Moderately,” and “Very much.” These responses were categorised as follows: (1) “Not at all” was recoded as “Never,” (2) “Not very much” and “Moderately” were collapsed into “Mild to Moderate,” and (3) “Very much” was recoded as “Severe.”
- *Geriatric Mental State Automated Geriatric Examination for Computer Assisted Taxonomy (GMS-AGECAT) [18]*. It is a semistructured questionnaire which applies a computer algorithm to identify nine diagnoses including organicity (probable dementia), across 5 levels of psychopathology from 0 (noncase) to 5 (severe case).
- *World Health Organization Disability Assessment Scale (WHODAS 2.0)*. WHODAS 2.0 was established as an international and cross-cultural method to assess severity of disability in patients [19]. WHODAS was developed by the International Classification of Functioning, Disability, and Health (ICF) to identify symptoms of disorders that hindered every day living. Disability levels measured by this assessment have good test-retest reliability with validation in 16 languages in 14 countries. WHODAS measures functioning based on six domains: cognition, mobility, self-care, getting along, life activities, and participations. Items were measured and computed using a specific scale: “None” (0), “Mild” (1), “Moderate” (2), “Severe” (3), and “Extreme” (4). Items in each domain were summed and weighted; then all six weighted scores were converted into a summary score ranging from 0 to 100 (where 0 indicates no disability and 100 indicates full disability).
- *Sociodemographic Questionnaire*. This included questions on age, gender, ethnicity, marital status, education, employment status, social support, and personal/family income.
- *Health Status Questionnaire*. It included a chronic conditions checklist that asked participants if they had been diagnosed by a doctor as having any chronic illness: specifically hypertension, diabetes, and transient ischemic attack.

### 2.2. Statistical Analysis

All statistical analyses of 10/66 protocols were carried out using the Statistical Analysis Software (SAS) System version 9.3. Frequencies and percentages were calculated for all categorical variables. Multiple logistic regression analysis was performed to study the sociodemographic correlates of pain. Association between pain and comorbid health conditions was established using multiple logistic regressions. Depression, hypertension, transient ischemic attacks, diabetes mellitus, and dementia were included as predictor variables for pain. Association of pain and sociodemographic characteristics with WHODAS 2.0 was also established using multiple logistic regressions.

## 3. Results

The prevalence of pain in the past month was observed to be 19.5% among people aged 60 years and above in Singapore (Table 1). Logistic regression analysis found that women (1.8 times) were more likely to be associated with pain as compared to males (Table 1). Pain reporting varied with ethnicity; Malay (1.4 times), Indian (2.4 times), and those belonging to other ethnicities (3.4 times) showed significant association with pain (Table 1). Compared to those with tertiary education, those who did not complete primary education (2 times) were more likely to be associated with pain (Table 1).

Pain was substantially associated with the presence of other comorbid illnesses like transient ischemic attacks (2.9 times), depression (2.8 times), and hypertension (1.8 times) (Table 2). Multiple linear regression analysis performed after being adjusted for sociodemographic variables and any chronic conditions showed that the risk of disability was significant among those experiencing pain (Table 3). Participants who stated frequency of pain as more than “2-3 days in a week” showed significant association with disability (*p* < 0.001) as compared to those who stated “never” (Table 3). In terms of severity of pain, participants who expressed having “severe” (*p* < 0.0024) and “moderate” (*p* < 0.0022) pain showed substantial association with disability as compared to “mild” (Table 3). Also, restriction in daily activities due to pain was significant among those who reported “mild to moderate” (*p* < 0.0001) and “severe” (*p* < 0.0001) pain as compared to those who stated that they “never” had pain (Table 3).

## 4. Discussion

The cross-sectional epidemiological study of older adults in Singapore established the prevalence of pain experienced in the past month as 19.5%, which is in line with previous research by Blyth et al. [20] on the adult Australian population. Few other large scale surveys documented the prevalence of pain among those aged 60 years and above, ranging from as low as 9.3% to 73.6% at the higher end, respectively [21–23].

Women participants of our study were more likely to report chronic pain, a finding which is in line with prior research [24–26]. This might be due to greater sensitization to pain and its reporting because of underlying biological mechanisms or psychosocial factors specific to female gender [27, 28] such as sex role beliefs, pain coping strategies, and sex hormones which are known to affect pain responses [29, 30].

The study also found significant differences in pain reporting by ethnicity. Our results suggest that those who belonged to other ethnic groups were more likely to report pain followed by Indian and Malay ethnicities as compared to Chinese ethnic group. Earlier work has examined ethnic and cultural issues with respect to pain perception and tolerance [31]. Each person has preconceptions and personal beliefs about pain that are based on cultural, sociologic, and ethnic issues [32, 33]. Also, it is commonly believed that different ethnic groups have different pain tolerances, but this is poorly quantified [34]. Social customs within an ethnic population may dictate the extent of pain expressed. But to date there is a dearth of knowledge with regard to pain perception amongst the elderly aged 60 years and above in an Asian population.

Our findings are in accordance with prior research indicating that the elderly with lower levels of education were more likely to report pain as compared to those with higher levels of education [35]. Lower levels of education are often related to lower socioeconomic status which may lead to limited access to available resources and rationed utilisation of health care services resulting in poor health conditions [36]. Along with this, impaired communication with the health professionals due to lower level of education may also be a barrier in following the treatment plan [37]. Also, few mechanisms that could explain this association include variations in behavioural and environmental risk factors by educational status, differences in occupational factors, adaptation to stress, and compromised health stock among people with lower levels of education [38].

As hypothesized, pain was significantly associated with the presence of other comorbid illnesses among the study population. We observed that those suffering from comorbidities such as transient ischemic attacks (TIAs), hypertension, and depression were at a higher risk of experiencing pain which is in accordance with previous research [9, 10]. TIA and hypertension are often observed together due to the similarity in their risk factors and clinical features. Symptoms such as dizziness, vertigo, temporary blindness, and lack of coordination among those diagnosed with these illnesses make them prone to falls causing physical injury and pain [39]. Also, functional weakness restricts activity which may give rise to overstraining and pain. The association between pain and depression may be bidirectional. Few authors believe that recurrent chronic pain increases depressive symptoms whereas others advocate that periodic depressive episodes multiply the risk of pain development [40].

Prior studies have observed that restriction of activity is directly proportional to frequency and severity of pain in the presence of any comorbid condition [10, 41]. However, a study among the elderly experiencing pain indicated that restriction of activity signifies an effective pain-reduction approach [42] whereas another study reported that activity restriction is not an effective measure to manage pain [43].

The strengths of the study include its large sample size which was drawn from a representative sample population, administration of questionnaires in local dialects, implementation of stringent quality control measures, and practicing multiple attempts of contact to get good response rate. Our study incorporated reliable pain instrument, similar to other widely used questionnaires having a potential to provide a complete picture of the complex relationship between pain, comorbidities, and functional disability as compared with what has been published to date [10].

However, certain limitations need to be considered while interpreting the study findings. The response rate of our study was acceptable (65.6%); however, the probability of pain among those who refused to participate in our study could have been higher. Reluctance to report pain could also be possible by respondents which may be motivated by fear due to uncertain aetiology of persistent pain and diagnostic and prognostic consequences [44, 45]. The cross-sectional nature of our study restricted our ability to determine time course or infer causality between chronic pain and associated factors. Even though we have theorized that pain predicts disability, the possibility of this relationship being bidirectional cannot be denied.

## 5. Conclusion

In conclusion, it is noticed that pain is independently associated with older adult population in Singapore. Based on our study and the published research, it is reasonable to suggest that improving pain management and treatment of comorbidities may shift the threshold of disability among the elderly. Even a small increase in treatment success may reduce the degree of pain, keeping older adults independent for longer periods in the community. Studies are also needed to ensure that more aggressive pain management is not associated with unanticipated functional loss or other side effects.

## Figures and Tables

**Table 1:** Sociodemographic correlates of pain.

Variables	*N* (participants who reported pain)	%	Odds ratio^*∗*^	95% CI	*p* value
	**560**	19.49			
*Age group *					
60–74	**331**	18.2	**Ref**		
75–84	**151**	23.8	1.3	(0.9, 1.9)	0.126
85+	**78**	25.9	1.3	(0.8, 2.9)	0.286

*Gender *					
Males	**183**	15.2	**Ref**		
Females	**377**	23.0	1.8	(1.2, 2.7)	**0.003**

*Ethnicity*					
Chinese	**163**	17.9	**Ref**		
Indians	**222**	30.9	2.4	(1.8, 3.0)	**<0.001**
Malay	**162**	23.0	1.4	(1.0, 1.9)	**0.019**
Others	**13**	39.0	3.4	(1.6, 7.5)	**0.002**

*Marital status*					
Married/cohabiting	**319**	18.8	**Ref**		
Divorced/separated	**32**	26.3	1.6	(0.8, 2.9)	0.159
Never married	**26**	16.7	0.9	(0.5, 1.7)	0.729
Widowed	**183**	21.1	0.7	(0.5, 1.1)	0.136

*Education*					
Completed tertiary education	**44**	14.6	**Ref**		
None	**110**	22.8	1.5	(0.8, 2.9)	0.199
Completed primary education	**144**	16.7	1.2	(0.7, 2.1)	0.547
Some, but did not complete primary education	**143**	25.5	2.0	(1.1, 3.6)	**0.018**
Completed secondary education	**117**	16.7	1.2	(0.7, 2.1)	0.629

*Employment *					
Paid work (part-time and fulltime)	**118**	15.7	**Ref**		
Homemaker	**209**	22.6	1.0	(0.6, 1.6)	0.970
Retired	**221**	21.3	1.3	(0.1, 1.1)	0.138
Unemployed	**5**	16.3	1.1	(0.3, 4.4)	0.907

^*∗*^Odds ratio was derived from logistic regression by controlling for all the correlates.

**Table 2:** Relationship between overall pain and chronic health conditions.

Chronic health conditions	*n*	%	Odds ratio^*∗*^	95% CI	*p* value
*Hypertension*					
No			Ref		
Yes	402	23.3	1.8	1.3, 2.4	**0.001**

*Diabetes mellitus*					
No			Ref		
Yes	196	23.4	1.2	0.9, 1.7	0.217

*Transient ischemic attacks*					
No			Ref		
Yes	22	40.1	2.9	1, 8	**0.045**

*10/66 dementia*					
No			Ref		
Yes	59	27.0	1.1	0.6, 2	0.669

*GMS depression*					
No			Ref		
Yes	233	37.6	2.8	2, 4	**0.001**

*Any of the above chronic conditions*	504	23.3	2.8	1.8, 4.3	**0.001**

^*∗*^Multiple logistic regression adjusted for sociodemographic variables.

**Table 3:** Relationship between pain and WHODAS disability score.

Variables	Beta coefficient^*∗*^	Std error	95% CI		*p* value
			Lower limit	Upper limit	
*Frequency of pain*					
2-3 days a week	11.1	1.4	8.3	13.9	**<0.001**
2-3 times a month	1.3	1.0	−0.7	3.3	0.218
Never	Ref	Ref	Ref	Ref	·

*Severity of pain*					
Severe	9.5	3.1	3.4	15.6	**0.002**
Moderate	6.5	2.1	2.3	10.6	**0.002**
Mild	Ref	Ref	Ref	Ref	·

*Restriction of activity due to pain*					
Severe	20.9	3.8	13.4	28.5	**<0.001**
Mild to moderate	8.2	2.0	4.3	12.1	**<0.001**
Never	Ref	Ref	Ref	Ref	·

^*∗*^Multiple linear regression analysis adjusted for sociodemographic variables and presence of any comorbid condition.
